# Evaluating the risk factors for Lubinus SP II femoral stem fractures: A case series of 5 primary total hip arthroplasty patients with a mean follow-up of 7.5 years

**DOI:** 10.1177/2050313X251366366

**Published:** 2025-08-28

**Authors:** Alexis Panzures, Saim Faisel, Akkhash Sivakumar, Syed Ashoor Hasan Kazmi, Gareth Turnbull, Andy Ballantyne, Muhammad Adeel Akhtar

**Affiliations:** 1 University of Edinburgh, Edinburgh, UK; 2 Akhtar Saeed Medical and Dental College, Lahore, Pakistan; 3 Victoria Hospital, Kirkcaldy—NHS Fife, Scotland, UK; 4 University of St Andrews, St Andrews, UK

**Keywords:** total hip arthroplasty, THA, femoral stem fracture, revision arthroplasty, case series

## Abstract

The reported rate of femoral stem fracture after total hip arthroplasty (THA) varies between less than 0.1 and 3.4%. The study aimed to evaluate the incidence of Lubinus SP II femoral stem fracture in our population and associated risk factors, and to examine clinical outcomes following revision THA for SP II stem fracture. 4244 primary THAs incorporating the anatomic femoral stem were identified within our institution from a prospectively compiled arthroplasty patient database. 5 patients presented with a broken Lubinus SP II anatomical hip stem. Immediately postoperatively, the operating consultant submitted intraoperative data detailing surgical approach, head size, and components used. Patients were reviewed 6 weeks postoperatively in an orthopedic clinic, then followed up at a dedicated orthopedic audit clinic at 6 months, 1, 3, and 5 years postoperatively, and data were collected prospectively. Postoperative complications were recorded at each follow-up visit. The incidence of stem fracture was 0.1% (5/4240) at a mean follow-up of 7.5 years. 3 were male, and 2 were female. The mean age was 63.8 years (range, 53–72, SD = 7.4). The mean weight was 109 kg (range, 88–128; SD = 14.2). The mean BMI was 36.5 kg m^−1^ (range, 32.5–41.0, SD = 3.08). The mean time from primary THA to fracture was 6.4 years. The mean size of the cement restrictor (indirectly suggesting the femoral canal diameter) was 13.6 mm (range, 12–15, SD = 1.1). The implant neck angle used was 117 in 4 patients and 126 in 1 patient. The mean stem position in varus was −2.2 (range, −6–0, SD = 3.0). 4 fractures (80%) occurred at mid-stem and 1 (20%) distally with −6 degrees varus and a 15 mm cement restrictor. To minimize stem fracture risk, we recommend using as large a size stem as possible after sequential reaming in tight femoral canals and avoiding stem downsizing along with holistic postoperative management.

## Introduction

The reported rate of femoral stem fracture after total hip arthroplasty (THA) varies between less than 0.1% and 3.4%.^1–4^ With an aging population, increasing demand for THA^5^ is expected to result in growing stem fracture prevalence.^6,7^ However, there is limited data describing risk factors associated with femoral stem fractures in individual implant types. Understanding these risk factors could prevent these fractures through appropriate patient selection and surgical decision making.

Femoral stem fracture involves fracture of the implanted stem with or without fracture of the surrounding bone (periprosthetic fracture), and is generally thought to occur due to fatigue of the implant generated by unfavorable surrounding biomechanics that lead to mechanical overload.^8^ Stem fracture is a devastating complication for patients, as it usually mandates the need for revision THA, with removal of the broken stem frequently involving additional invasive steps.^9^ Patient, implant, and surgical risk factors are all thought to contribute to stem fracture risk. Patient risk factors include, but are not limited to, increased body mass index (BMI), high amounts of physical activity, younger age, male sex, and reduced proximal bone stock (often in the context of undergoing revision THA). Implant factors, including the use of modular stems, different stem designs, inferior stem materials, and implant etching, can also increase risk. Surgical factors noted to increase risk include varus implant insertion, inadequate cement mantle, and undersizing of the prosthesis.^10–12^

**Figure 1.**
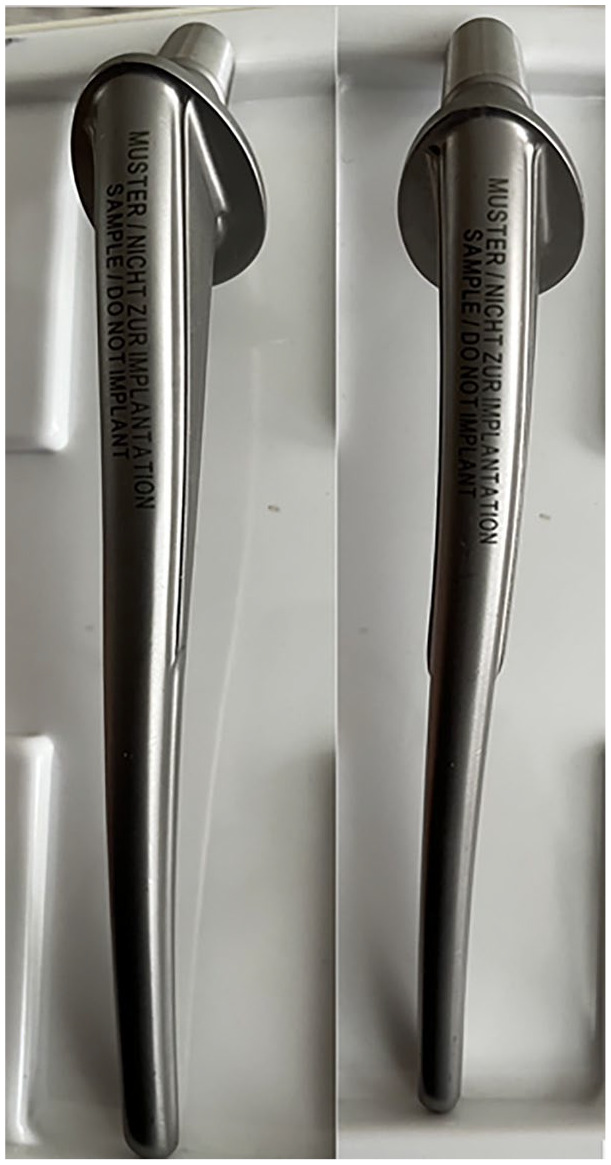
Lubinus SP II stem—orthogonal views.

The Lubinus SP II implant (see Figure 1) was first introduced in 1982 as the first anatomical stem with built-in neck anteversion, and includes a number of design features aimed at improving stem survivorship.^13^ Recently, this anatomical stem demonstrated excellent survivorship and negligible rates of periprosthetic fractures at a mean of 12 years,^14^ and over 20 years^15^ following primary THA. It is composed of a chrome cobalt alloy that is corrosion resistant^16^ while having high mechanical strength.^17^ The S-shape design of the stem resembles the anatomic curvature of the human femur, helping to facilitate central positioning of the stem in the medullary canal, equal distribution of cement around the stem, and also maximizes stem resistance to rotational forces.^18^ Physiological anteversion helps delay eventual aseptic loosening via optimum bearing of stress on stem torsion.^19^ A calcar collar helps to reduce the rate of fracture by allowing physiological force transmission on to the femur resulting in a uniform distribution of stress without any focal stress point.^20, ^21^^ The stem has 3 standard stem lengths (130, 150, 170 mm) and 4 revision stem lengths (200–350 mm); 4 head sizes (0, −3.5, +4 and +8 mm); and 3 femoral neck angles (caput-collum-diaphyseal (CCD) angles 117°, 126°, and 135°). To reduce stem fracture risk, insertion of larger rather than smaller femoral stems is recommended where possible, to increase resistance to mechanical fatigue and cantilever forces applied to the femoral stem.^22^ To achieve this with the SP II stem, it is recommended to (1) avoid downsizing the cemented SP II stem from the stem size the femur is prepared for; and if necessary, (2) use flexible reamers in patients with very tight femoral canals, to allow access for larger broaches for femoral preparation.^22^

Despite the long-term survival of the implant in primary THA,^18^ fractures of the Lubinus SP II stem have not been reported. The primary aim of this paper was to evaluate the incidence of SP II femoral stem fracture in our cohort and identify associated patient and surgical risk factors. The secondary aim included examining clinical outcomes following revision THA for SP II stem fracture.

### Patients and methods

Between January 1998 and December 2018, 4244 primary THAs incorporating the anatomic femoral stem (Lubinus SP II) were performed within our institution. These patients were identified from a prospectively compiled arthroplasty patient database administered by a dedicated audit nurse. Preoperative data were collected prospectively, including patient demographics and BMI.

Immediately postoperatively, the operating consultant submitted intraoperative data detailing surgical approach, head size, and components used. Patients were reviewed 6 weeks postoperatively in an orthopedic clinic by the operating consultant. They were then followed up at a dedicated orthopedic audit clinic by two specialist nurses at 6 months, 1, 3, and 5 years postoperatively, and data were collected prospectively. Postoperative complications were recorded at each follow-up visit.

Five patients presented between July 2007 and March 2018 with broken Lubinus SP II anatomical hip stems used for primary THA. Risk factors for stem fracture, including BMI, patient’s weight, size, and position of the stem and site of stem fractures, were recorded from clinical notes and review of the x-rays. The stem alignment was calculated by finding the angular deviation of the stem tip from the anatomical femoral axis; a clockwise rotation of the implant axis corresponds to a varus orientation, and a counterclockwise rotation corresponds to a valgus orientation. Mechanical and metallurgical analysis was considered but deemed unavailable. Outcomes following revision THR for these stem fractures were reported.

Table 1 provides an overview of the patients identified in this case series.

**Table 1.** Demographic details.

Patient number	Age	Sex	Height (cm)	Weight (kg)	BMI (kg m^−1^)	Time to follow-up (years)
1	61	M	184	110	32.5	4.9
2	53	F	176	107	37	6.5
3	69	M	176	128	41	6.2
4	64	M	177	111	35.3	5.9
5	72	F	155	88	36.6	14.1

*Note.* F = Female; M = Male.

### Surgical technique

THA implants^23–25^ and surgical approaches^26^ have been described in detail. The Lubinus SP II surgical implant and technique are described in detail elsewhere,^14^ and summarized as follows. The Lubinus SP II stem is specifically designed for cement fixation and is used with a metal-on-polyethylene acetabular cup (although it can be used with other cups). All patients underwent primary unilateral THA by 1 of 12 consultant orthopedic surgeons at our practice with an interest in lower limb arthroplasty. In the lateral decubitus position, a modified Hardinge or posterior approach was used. Femoral component stems were used throughout. Preoperative templating and intraoperative adjustments were made to achieve a balanced hip. The femur underwent broaching, lavage, and then a cement restrictor was inserted. Third-generation cementing technique was used (involving pulsatile jet lavage, retrograde cement application, and 3-phase pressurization before stem insertion) prior to stem insertion. Cemented acetabular components were similarly implanted. Acetabular implants included cemented Lubinus acetabular components (Waldemar LINK GmBh& Co, Hamburg, Germany), Pinnacle uncemented acetabular components (DePuy Synthes), and Mathys RM Pressfit acetabular components (Mathys Ltd, Bettlach, Switzerland). Palacos R & G cement was used for cementation (Heraeus Kulzer GmbH; Heraeus Medical GmbH). Unless contraindicated or failed, all patients underwent spinal anesthesia. Drains were not used. Antibiotic prophylaxis was a single intravenous dose of 1 gm of ceftriaxone (unless contraindicated). Standardized venous thromboembolism prophylaxis was in the form of 10 mg rivaroxaban once daily for 35 days. Patients then completed standard postoperative care and rehabilitation protocol.

#### Data analysis

Statistical analysis was performed with SPSS V27.0.0.0 (IBM Corp., Armonk, NY, USA). Quantitative variables, including age, height (m), weight (kg), BMI (kg m^−1^), and cement restrictor size (mm), were expressed as mean ±SD. Categorical variables, including side, model type, stem size, offset details, and type of stem fracture, were expressed as proportions and percentages (%).

#### Ethics, study design, and checklist

Institutional ethical approval was not required for this observational study reporting the incidence of femoral stem fractures. This paper follows the Case Report Guidelines (CARE) Checklist^27^ for case reports.

## Case presentations

Key information regarding patient demographics and follow-up is presented in Table 1. The incidence of stem fracture was 0.1% (5/4240) at a mean follow-up of 7.5 years. The paper included 5 patients. 3 were male, and 2 were female. All 5 patients were White (Scottish). The mean age was 63.8 years (range, 53–72, SD = 7.4). The mean height was 1.74 m (range, 1.55–1.84, SD = 0.11). The mean weight was 109 kg (range, 88–128, SD = 14.2). The mean BMI was 36.5 kg m^−1^ (range, 32.5–41.0, SD = 3.08). Patients 2, 3, and 4 had an increase in BMI after hip replacement surgery from 34 to 37 kg m^−1^, from 35 to 41 kg m^−1^, and from 31 to 35.3 kg m^−1^, respectively.

**Figure 2.**
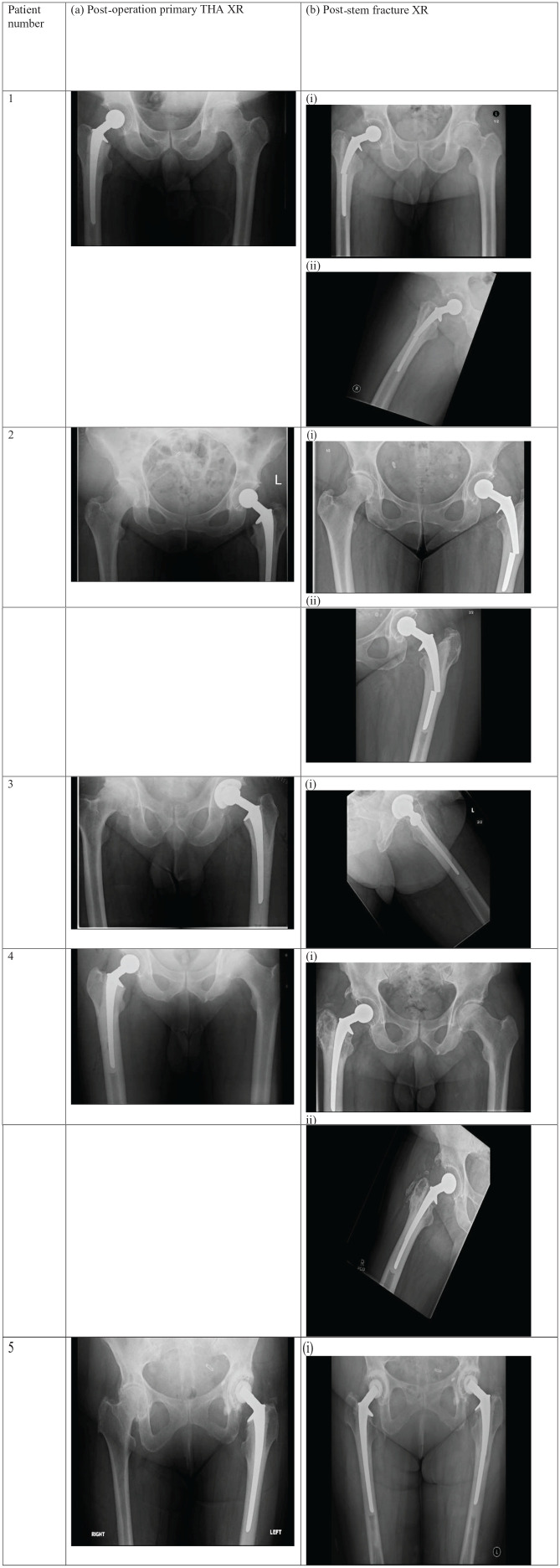
X-ray images following primary THA and subsequent stem fracture. *Note.* X-rays following primary THA (a) and femoral stem fracture (b) (Patients 1–5). (i) Anteroposterior view. (ii) Lateral view congruent with the fractured stem (see Table 2).THA = total hip arthroplasty.

Primary surgery details (including side of THA, type of fixation for hip replacement implants, cement restrictor size, stem size, offset and position, time to stem fracture, and type of stem fracture) are summarized in Table 2. Patient x-rays taken before and after femoral stem fracture are presented in Figure 2. The mean time from primary THA to fracture was 6.4 years. 4 (80%) patients experienced a fracture with hybrid THA, while only 1 (20%) had cemented THA. The mean size of the cement restrictor (indirectly suggesting the femoral canal diameter) was 13.6 mm (range, 12–15, SD = 1.1). The stem size was 01 in 2 patients and 1 in 3 patients. 2 patients receiving 01 stems had downsizing of the final implant after a size 1 rasp was used. The implant neck angle used was 117 in 4 patients and 126 in 1 patient. The mean stem position in varus was −2.2 (range, −6–0, SD = 3.0). 2 stems were in varus −5 and −6. 2 patients had an XL neck used (Patients 2 and 3). All stems were 150 mm long. Interestingly, 4 fractures (80%) occurred at the mid-stem and only 1 (20%) at the distal stem. Patient 4 sustained a distal stem fracture with −6 degrees varus and a 15 mm cement restrictor.

**Table 2.** Primary THA details.

Patient number	Side (L/R)	Cemented/ uncemented/ hybrid	Cement restrictor size (mm)	Stem size	Stem position (varus) (°)	Offset details	Time to implant fracture (years)	Type of stem fracture
1	R	Hybrid	14	01	0	117	3.8	Mid stem B1
2	L	Hybrid	14	01	0	126	6.3	Mid stem B1
3	L	Hybrid	12	1	−5	117	5.5	Mid stem B1
4	R	Hybrid	15	1	−6	117	4.0	Distal 3^rd^ stem
5	L	Cemented	14	1	0	117	12.3	Mid stem B1

*Note.* L: Left; R: Right.

THA = total hip arthroplasty.

All patients were revised to uncemented, diaphyseal fixed implants for revision THA between November 2020 and February 2023. At the last clinical follow-up one patient reported leg length discrepancy, while the remainder were progressing well without any reported complications.

## Discussion

We reported the incidence of Lubinus SP II anatomical hip stem used for THA in our cohort over the last 20 years to be 0.1%, which is less than other common primary THA stem implants.^28^ This study set out to understand risk factors associated with femoral stem fractures following primary THA with the Lubinus SP II Femoral anatomical implant. The outcomes focused on demographic details of patients and primary surgery details (including size and length of the stem, position of the stem, and the use of cement). The most important finding was that all patients in our cohort demonstrated one or more risk factors that predisposed them to a femoral stem fracture. All of the patients experiencing femoral stem fracture were obese (BMI > 30 kg m^−1^) with tight femoral canals (hence, small diameter stems were used). Most patients had hybrid THA, and stems in varus alignment were common in our cohort.

High BMI is well associated with increased risk of complications following THA, including infection, delayed wound healing, periprosthetic fracture, and reoperation.^29^ High BMI and patient weight were found to be risk factors for femoral stem fracture in our cohort, with all patients experiencing stem fracture clinically obese (BMI > 30 kg m^−1^) and having a mean weight of 109 kg.^30^ These findings are similar to previous case series,^31–33^ with our data supporting these findings for the Lubinus SP II stem. It is plausible that the altered loading dynamics, compromised bone quality often associated with obesity, and potential challenges in achieving optimal prosthetic alignment collectively predispose these individuals to femoral stem fractures.^34–36^ Patient weight often changed over the duration of follow-up, which may have altered their risk of fracture following the primary THA.^37^

The mean time from primary THA to stem fracture was 6.4 years. 80% of patients experienced fractures within an 11-year *at-risk* period during which the majority of fractures have previously been suggested to occur.^38^ Interestingly, Patient 5 was the last to experience femoral stem fracture following primary THA despite being female and in the post-menopausal age group. It is speculated that this is due to decreased activity level,^39^ and decreased energy expenditure when compared to younger patients^40^ decreasing both the fracture and falls risk.

In most patients (80%), femoral stem fractures occurred at the mid-stem position. Mid-stem fractures are likely caused by cantilever bending stresses,^41^ with irrelatively greater stability and fixation in the distal stem compared to the proximal stem, causing fatigue failure. A common risk factor in our cohort was varus alignment in primary THA. The stems of Patients 3 and 4 were in varus −5° and −6°, respectively, which increases stress on the femoral stem.^34^, ^35^ This clinically presented as an earlier stem fracture, and an increased risk of femoral stem fracture in varus-aligned stems in keeping with recent literature.^35^, ^38^, ^39^, ^41–43^ Notably, Patient 4 was the only patient to sustain a distal stem fracture with −6° varus and a 15 mm cement restrictor. Contrastingly, 3 (60%) primary stems were in a neutral position (0 varus), reflecting a multifaceted aetiology of fracture. Similar to current literature, a large offset was associated with fractures in our cohort due to increased leverage and subsequent stress on the implant.^28^, ^44^, ^45^ Patient 5 with a cemented femoral stem had the longest time to fracture (12.3 years) in contrast to hybrid models, although the mechanism of this is unclear.

It is important to recognize the limitations of this study. While over 4000 stems were included, this is a small cohort of only 5 stem fractures, limiting more detailed analysis of risk factors. While all patients experienced femoral stem fracture following primary THA, there was significant heterogeneity in patient demographics and primary surgery details. In addition, metallurgical data for our cohort were not obtained or analyzed, which may have affected prosthesis wear and longevity. It also remains possible that some patients were lost to follow-up on leaving our institutional catchment area, although national radiographic archives were searched for episodes of patients presenting to other hospitals to mitigate this as much as possible. Despite the study limitations, this represents the largest analysis of SP II stem fracture incidence over long-term follow-up. To our knowledge, this study is the first to identify risk factors for fractured femoral stems of the Lubinus SP II implant resulting in revision THA. To add to a growing body of evidence identifying demographic and surgical risk factors leading to THA complications, and to further mitigate these factors, should not be underappreciated.

## Conclusion

Overall, we found the Lubinus SP II stem to have a fracture rate of 0.1% over 20 years. The patients in our cohort had a mean follow-up of 7.5 years following primary THA. All patients in our cohort had a BMI > 30 kg m^−1^. Varus stem alignment was also a risk factor for stem fracture. To minimize stem fracture risk, we recommend using as large a size stem as possible after sequential reaming in tight femoral canals and avoiding stem downsizing along with holistic postoperative management (including weight loss where possible). The authors recommend implant-specific surveillance in future research.
